# Protein and mRNA expression of uPAR and PAI-1 in myoepithelial cells of early breast cancer lesions and normal breast tissue

**DOI:** 10.1038/sj.bjc.6601990

**Published:** 2004-06-29

**Authors:** R Hildenbrand, N Arens

**Affiliations:** 1 Pathologisches Institut, Universitätsklinikum Mannheim, Universität Heidelberg, Theodor-Kutzer-Ufer 1-3, Mannheim 68167, Germany

**Keywords:** uPAR, PAI-1, DCIS, myoepithelial cells

## Abstract

Myoepithelial cells (MEs), which surround ducts and acini of the breast glands, exhibit an anti-invasive phenotype and form a natural border separating proliferating tumour cells of ductal carcinoma *in situ* (DCIS) from basement membrane (bm) and underlying stroma. Invasion requires penetration of these host cellular and extracellular matrix barriers. This destruction is caused by proteolytic activity of tumour cells and host bystander cells. There is substantial evidence that high concentrations of the urokinase plasminogen-activating system are conducive to tumour cell spread and metastasis. Prompted by the conspicuous absence of studies examining the role of the ME in breast cancer progression, we studied the expression of the urokinase plasminogen activator receptor (uPAR) and plasminogen activator inhibitor type-1 (PAI-1) in MEs of 60 DCIS samples. Our results show that nearly all MEs of DCIS and normal breast glands exhibit the uPAR antigen, whereas the PAI-1 antigen was mainly expressed in MEs of high-grade DCIS. In one intermediate DCIS numerous ducts showed an incomplete myoepithelial layer expressing uPAR and PAI-1. We conclude that uPAR in MEs may be necessary to attach them to the bm by uPAR/vitronectin (Vn) interaction. The strong expression of PAI-1, which is known to resolve the uPAR/Vn binding, may be involved in the detachment of MEs of DCIS. Although the role of PAI-1 acting as cell detachment factor could not be demonstrated in our study, we speculate that the loss of the anti-invasive ME layer in DCIS may be triggered by PAI-1 and could be an early sign of subsequent tumour cell infiltration.

Ductal carcinoma *in situ* (DCIS) is a proliferation of malignant epithelial cells within the ductulolobular system of the breast that shows no evidence of invasion through the basement membrane (bm) into the surrounding stroma. For tumour progression and metastasis cancer cell invasion is necessary (Silverstein, 1998). The growth and development of invasive breast cancer is determined not only by the specific oncogenic or tumour suppressor alterations occurring within the malignant cell itself, but also by paracrine regulation exerted by many host bystander cells including fibroblasts, macrophages, and endothelial cells. One host cell type, however, the myoepithelial cell (ME), has not been studied in depth. Myoepithelial cells *in vivo* surround the ducts and acini of the breast and contribute to the synthesis of a surrounding bm. This anatomic relationship suggests that MEs may exert significant paracrine effects on breast epithelium, which regulate the progression of DCIS to invasive breast cancer (Sternlicht *et al*, 1997). Tumour cells cross host cellular and extracellular matrix barriers during tumour invasion and metastasis by attachment to and interaction with components of the bm and the extracellular matrix and by cellular proteolysis (Schmitt *et al*, 1997).

In retrospective studies, pathologists have noted an approximate 25% incidence of progression of DCIS to invasive cancer over a 6- to 10-year period (Page *et al*, 1982), but whether genetic events in DCIS or paracrine events governed by the myoepithelial or other host cell influence this progression is not known. It is believed that MEs are an important paracrine regulator of breast carcinoma progression and that MEs exert an anti-invasive role on the progression of DCIS. The high constitutive expression of the tumour suppressor maspin and diverse proteinase inhibitors, accompanied by low levels of proteinase expression provide support for this anti-invasive role for the ME (Sternlicht *et al*, 1997). It is not known whether a loss of the ME layer in DCIS is an early sign of subsequent invasion.

Determination of components of the plasminogen activator system in breast cancer is an important issue to address since there is substantial evidence that high concentrations of proteolytic factors in primary breast cancer tissue are conducive to tumour cell spread and metastasis (Jänicke *et al*, 1990). Penetrating tumour cells focus on the proteolytic activity of the serine protease urokinase-type plasminogen activator (uPA) secreted by tumour cells or surrounding stromal cells to the cell surface through a receptor for uPA (uPAR, CD87), thus facilitating extracellular matrix degradation.

In addition to a role in localising uPA proteolytical activity to the cell surface, the uPAR also interacts with integrins such as vitronectin (Vn) and thus facilitates cell–matrix interactions (Wei *et al*, 1994). It was demonstrated by Wei *et al* (1996) that the capacity of uPAR to act as an adhesions receptor depends as well on a functional and physical association with integrins. Urokinase plasminogen activator receptor forms complexes with activated integrins, presumably utilising integrin connections to the cytoskeleton to promote stable adhesion to Vn mediated by the distinct binding site on uPAR. Urokinase plasminogen activator receptor/Vn interaction can be enhanced by uPA and attenuated by the PAI-1, which binds to the somatomedin B domain of Vn (Deng *et al*, 1996). In addition, uPAR is capable of modulating cell adhesion by activating cells directly via a G-protein-coupled receptor (Liu *et al*, 2002), by sequestering caveolin (Wei *et al*, 1999), and by affecting intracellular signalling events (Nguyen *et al*, 1999). Thus, uPAR is an important regulator of the adhesive behaviour of cells.

In previous studies, we have observed that components of the urokinase system are found not only in invasive breast cancer cells but also in MEs, macrophages, fibroblasts and tumour cells of DCIS (Hildenbrand *et al*, 1998, 2000). This suggests that uPAR expression of MEs, located in this strategically important position, may be involved in uPA/PAI-1-dependent tumour cell invasion. This prompted us to perform this study on DCIS with a special look on the expression of the urokinase system in MEs.

## MATERIALS AND METHODS

In all, 60 patients with DCIS were enrolled in the study. Tissue was obtained by surgery (either by breast preservation (*n*=56) or mastectomy (*n*=4)). The patients were on average 54.6±10.3 (mean±s.d.) years of age (median 53 years, range 30–79 years). Histological types were categorised according to their architectural patterns. Four main patterns were observed: comedo-, micropapillary-, cribriform- and solid type. Most DCIS showed mixed patterns. The ‘Van Nuys (VN) Classification’ for DCIS, introduced in 1995 by Silverstein and co-workers, was used in this study.

Group I (*n*=20) consisted of non-high-grade DCIS without comedo-type necrosis, group II (*n*=16) of non-high-grade DCIS with comedo-type necrosis and group III (*n*=24) of high-grade DCIS, irrespective of comedo-type necrosis. For all of the cases, the mean tumour diameter was 32.2±11.2 mm (median 31 mm, range 3–64 mm). In all, 12 cases with normal (nontumour, benign) breast tissue were also examined.

### Immunohistochemistry and double immunostaining

Tumour specimens were formalin fixed and paraffin embedded. The tissue sections were stained by the APAAP method as described previously (Hildenbrand *et al*, 1998, 2000) by applying anti-uPAR monoclonal antibody (mAb) #3936 (American Diagnostica, Germany), chicken polyclonal antibody (pAb) HU277 and mAb IID7 (a kind gift from Dr. Magdolen, Luther and Schmitt, TU München and TU Dresden, Germany); pAb HU277 is directed to recombinant human uPAR (corresponding to amino acids 1–277 of uPAR) expressed in transfected CHO cells (Magdolen *et al*, 1995); mAb #3936 (IgG2a) to uPAR expressed by phorbol ester-stimulated promyeloid U937 cells; and mAb IID7 to human nonglycosylated uPAR polypeptide_1–284_ expressed in *Escherichia coli* (Luther *et al*, 1997). Consecutive tissue sections were stained with mAbs to alpha-actin (DAKO, Germany), anti-calponin (BioGenex, Germany), anti-uPA (American Diagnostica, Germany, #3688), anti-PAI-1 (American Diagnostica, Germany, #3785) and anti-Vn (mAb 892C, Innovex, Germany).

In all cases, a double staining was performed starting with the mAb IID7 (mAb anti-PAI-1) detected with the APAAP method, then proceeding with mAb anti-calponin detected with the streptavidin–biotin–POD method. For the second reaction, the DAB (diaminobenzidine)-staining kit (Leinco, k107, Germany) and ‘metal-enhancing solution’ was used. The immunostaining is in red (APAAP, neufuchsin) and black (streptavidin–biotin–peroxidase, DAB plus metal-enhancing solution), and the nuclei were counterstained with haematoxylin (blue colour). In each case negative controls were performed by substituting nonimmune antibodies (IgG) for mAb #3936, pAb HU277 and mAb IID7, respectively. In addition, the staining reaction was blocked by preincubation of pAb HU277 with an excess of CHO-uPAR_1–277_ prior to the staining reaction.

In 25 cases of DCIS and in 15 cases of normal breast tissue, a collagen type-4/Vn double staining was performed. The sections were incubated with mAb anti-Vn (Innovex Bioscience, Germany; dilution 1 : 50), washed and incubated with Texas Red sulphonyl chloride-conjugated rabbit anti-mouse IgG (Dianova, Hamburg, Germany; dilution 1 : 1000). Sections were then incubated with anti-collagen type-4 (DAKO, Hamburg, Germany; 100 *μ*l section^−1^) that had been biotinylated previously (ARK biotinylating-kit; DAKO, Hamburg, Germany). The biotin label was subsequently visualised with FITC-conjugated streptavidin (Vector Laboratories, Burlingame USA; dilution 1 : 250). Controls were incubated with nonimmune antibodies applied at the concentration as the primary mAb. In the controls, no specific immunolabelling was observed.

### *In situ* hybridisation

*In situ* hybidisation with fluorescein-labelled oligodeoxynucleotides was performed following the protocol of Hildenbrand *et al* (1998, 2000). For the detection of fluorescein-labelled oligodeoxynucleotides, the ‘Super Sensitive mRNA Probe Detection System’ (BioGenex, CA, USA) was used. The antisense oligodeoxynucleotides (Biometra, Germany) were complementary to nucleotides 121–150, 321–350, 521–550, 717–746 and 918–947 of uPAR mRNA and 181–210, 421–450, 661–690, 901–930 and 1081–1110 of PAI-1 mRNA (according to the nucleotide numbering of Accession number X51675 in the EMBL database).

### Laser capture microdissection of immunostained frozen sections for mRNA analysis

Serial frozen sections (4–8 *μ*m) were cut on a standard cryostat (Leica, Germany) with a clean blade. The unfixed tissue sections were immediately stored at −80°C until use. The frozen sections were thawed at room temperature for 30–60 s and immersed immediately in cold acetone (5 min). After fixation, the slides were rinsed briefly in phosphate-buffered saline (PBS, pH 7.4) and subjected to immunostaining. The immunostaining was performed with a modified DAKO staining kit (DAKO, Germany), a three-step streptavidin–biotin technique with prediluted monoclonal anti-smooth muscle actin (SMA) antibodies (anti-calponin, 1 : 80, BioGenex, Germany; and anti-alpha-SMA, 1 : 100, DAKO, Germany) optimised for very short staining times. The slides were incubated at room temperature with the primary and secondary antibodies and an alkaline-phosphatase-conjugated antibody for 90–120 s each and briefly rinsed in PBS between each step. After colour development with 0.04% 5-bromo-4-chloro-3-indolyl-phosphate (Roche, Mannheim, Germany) and 0.06% nitroblue tetrazolium (Sigma, München Germany) for 3–5 min and counterstaining with haematoxylin for 20 s, the sections were dehydrated in graded alcohols (15 s each) and xylene (2 × 2 min) and air-dried. After immunostaining and microscopic control of staining quality and tissue preservation, microdissection was performed using a laser capture microdissection microscope (Arcturus) equipped with an infrared laser. The dehydrated tissue section was overlaid with optically transparent caps, and cells were captured by focal melting through laser activation. After visual control of the completeness of dissection, the captured cells were immersed in denaturation solution (Fend *et al*, 1999).

### RNA extraction, reverse transcription (RT)

RNA was obtained from microdissected MEs (1500–2000 cells in each case) with the Micro RNA isolation kit (Stratagene, Germany). The RNA pellet was redissolved in 15 *μ*l sterile DEPC-treated water and incubated with 1 *μ*l of RNAse inhibitor (PE Applied Biosciences, Germany) and 20 U of DNAse I (GenHunter, Germany) for 2 h at 37°C in a total volume of 20 *μ*l. The amount and purity of RNA was calculated by using an Agilent Bioanalyser 2100.

The RT reaction was carried out in a total volume of 40 *μ*l: 1 × RT buffer (500 *μ*M deoxynucleotide triphosphates, 3 *μ*M random primers, 60 U of RNasin and 200 U of Superscript RNAse H^−^ (Invitrogen)). To this mixture, we added 1 *μ*g of total RNA. The reaction was allowed to proceed for 60 min at 37°C followed by 5 min at 95°C and a subsequent rapid cooling on ice. The cDNA was stored at −20°C until further use.

After re-extraction of RNA, RT was performed using 12 *μ*l of total RNA, 2.5 *μ*M random hexameres, 25 *μ*M dNTPs and 100 U of MMLV reverse transcriptase (Invitrogen, Germany). For each sample, a mock reaction without the addition of reverse transcriptase was performed.

### Qualitative and quantitative cDNA amplification

A measure of 1 *μ*l of the uPAR cDNA product was amplified in a thermal cycler (Autogene II Grant, Germany) for 35 cycles consisting of 60 s at 95°C, 90 s at 55°C and 3 min at 72°C. *Taq* polymerase was obtained from Perkin-Elmer Cetus and used according to the supplier's instructions. The following primers were based on the published uPAR (Casey *et al*, 1994) and PAI-1 (Ginsburg *et al*, 1986) sequence and synthesised by MWG Biotech, Germany: uPAR sense, 5′-CATGCAGTGTAAGACCAACG-3′; uPAR anti-sense, 5′-CTCTCACAGCTCATGTCTGATGAGCCAC-3′; PAI-1 sense, 5′-ACACCCTCAGCATGTTCATT-3′; and PAI-1 anti-sense, 5′-CTCGATCTTCACTTTCTGCA-3′.

The amplification products showed the expected size of 311 (uPAR) and 290 (PAI-1) base pairs. Appropriate negative controls including amplification of the mock RT reaction product were performed in each run. The polymerase chain reaction (PCR) products were separated on a 2% agarose gel stained with ethidium bromide.

Real-time PCR was performed in a LightCycler instrument using LC-Fast Start Reaction Mix SYBR Green I (Roche Diagnostics). Polymerase chain reaction amplification was carried out in a final volume of 10 *μ*l containing 1 *μ*l of cDNA sample; 1.2 *μ*l MgCl_2_ (25 mM); 0.2 *μ*l of PAI-1 primers (25 *μ*M each); and 1 *μ*l LC FastStart DNA Master SYBR Green I/Enzyme Mix (including *Taq* DNA polymerase, reaction buffer and deoxynucleotide triphosphate mixture). After an initial step of 10 min at 95°C (cDNA denaturation/HotStart-*Taq* polymerase activation), 40 amplification cycles were performed: 15 s at 95°C, 5 s at 58°C and 15 s at 72°C.

After PCR, a melting curve was created by increasing the temperature from 61 to 99°C with a temperature transition rate of 0.1°C s^−1^. Each PCR experiment was performed in triplicate.

For every LightCycler run, a standard curve was generated by the detection of the crossing point (CP) of each standard. The concentrations of unknown samples were then calculated by comparing their CPs to the standard curve.

## RESULTS

We have studied 60 different cases of DCIS, classified according to the ‘VNs Classification’ introduced by Silverstein *et al* (1995), for the expression and synthesis of uPAR by *in situ* hybridisation and immunohistochemistry. All tumour tissue sections were probed for the presence of uPAR mRNA by *in situ* hybridisation using fluorescein-labelled antisense oligodeoxynucleotides. With no exception, MEs, tumour cells, macrophages, fibroblasts and endothelial cells showed a positive reaction with the antisense probe (Figure 2G and H). Corresponding results were found in 12 cases with normal (nontumour) breast tissue. Epithelial cells, MEs as well as stromal cells showed a positive reaction with the antisense probe.

All types of DCIS were reacted with three different types of antibodies to uPAR (IID7, HU277, #3936; see Table 1a

**Table 1:** Anti-uPAR- and anti-PAI-1 immunoreactions in 60 cases of DCIS (a) and 12 cases of normal (nontumour) breast tissue (b)

	**Anti-uPAR IID7**	**Anti-uPAR HU277**	**Anti-uPAR 3936**	**Anti-PAI-1**
*(a) DCIS, n*=*60*^a^				
Myoepithelial cells	56 (18/14/24)	41 (15/12/14)	39 (14/11/14)	34 (2/8/24)
Tumour cells	56 (18/14/24)	50 (15/15/20)	31 (11/10/10)	56 (19/14/23)
Macrophages	60	60	60	60
Fibroblasts	60	60	60	60
Endothelial cells	56 (17/16/23)	38 (14/10/14)	0	60
				
*(b) Normal breast tissue, n*=*12*^b^				
Myoepithelial cells	10	8	5	1
Epithelial cells	10	7	4	1
Macrophages	12	12	12	12
Fibroblasts	12	12	12	12
Endothelial cells	7	4	0	12

**Figure 1:**
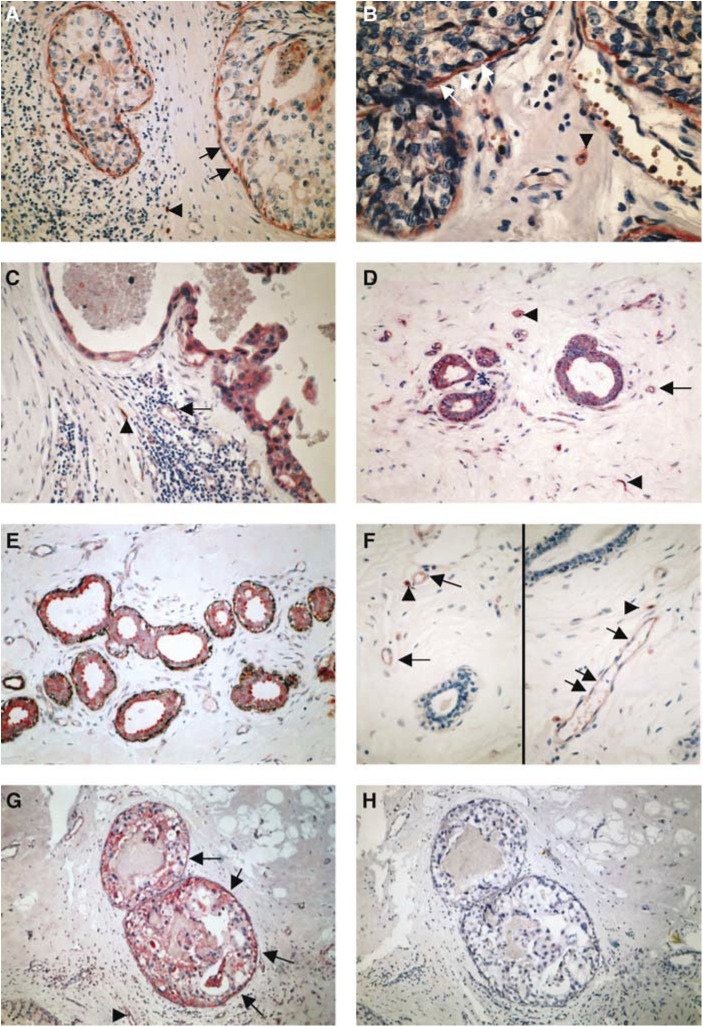
(**A**) Anti-uPAR HU277 immunoreaction of a high-grade DCIS. Myoepithelial cells show a strong staining (arrows) and tumour cells only a faint immunoreaction. Macrophages (arrowhead) are also positive. (**B**) Anti-uPAR IID7 immunoreaction of a high-grade DCIS. Myoepithelial cells show a strong immunoreaction (arrows) and tumour cells and endothelial cells are negative. Macrophages express the uPAR antigen (arrowhead). (**C**) Anti-PAI-1 immunoreaction of a high-grade DCIS. Tumour cells, endothelial cells (arrow) and stromal cells (arrowhead) are positive. The myoepithelial cell layer is absent; in corresponding tissue sections no MEs in this duct were observed using anti-calponin and anti-SMA antibodies. (**D**) Normal (nontumour) breast tissue stained for anti-uPAR IID7; MEs, epithelial cells and endothelial cells (arrow) show a strong immunoreaction, stromal cells (macrophages and fibroblasts) (arrowheads) are also positive. (**E**) Double staining of normal (nontumour) breast tissue stained for anti-uPAR IID7 (red colour) and anti-SMA (black colour); MEs show an immunoreaction for both anti-uPAR and anti-SMA. (**F**) Normal (nontumour) breast tissue stained for anti-PAI-1; in both images, the ducts (MEs and epithelial cells) are negative, whereas endothelial cells (arrows) and stromal cells (arrowheads) show positive immunoreactions. (**G**, **H**) Nonisotopic *in situ* hybridisation using fluorescein-labelled oligodeoxynucleotides complementary to PAI-1 mRNA in a non-high-grade DCIS with necrosis; (**G**) antisense probe: a distinct reaction in MEs (arrows) and tumour cells, stromal cells and endothelial cells (arrowhead) is observed; (**H**) no reaction is seen with the sense probe.

uPAR=urokinase plasminogen activator receptor; PAI-1=plasminogen activator inhibitor; DCIS=ductal carcinoma *in situ*. For anti-uPAR immunoreaction three different antibodies were used: mAb IID7, pAb HU277 and mAb 3936.

a The VNs Classification for DCIS (*n*=60) was used in this study: group I (VN G1, *n*=20) consisted of non-high-grade DCIS without comedo-type necrosis; group II (VN G2, *n*=16) of non-high-grade DCIS with necrosis; and group III (VN G3, *n*=24) of high-grade DCIS, irrespective of comedo-type necrosis. Behind the number of positive cases the results of group I, -II and III are given within parantheses (group I/group II/group III).

b Immunohistological results of 12 cases with normal (nontumour) breast tissue.

). In 56 of the cases (18 grade 1; 14 grade 2; 24 grade 3) MEs were stained by mAb IID7, 41 of those specimens (15 grade 1; 12 grade 2; 14 grade 3) reacted with pAb HU277 and 39 of those (14 grade 1; 11 grade 2; 14 grade 3) showed immunoreactivity of MEs with mAb #3936 (Figures 1A, B

and 2C, D

**Figure 2:**
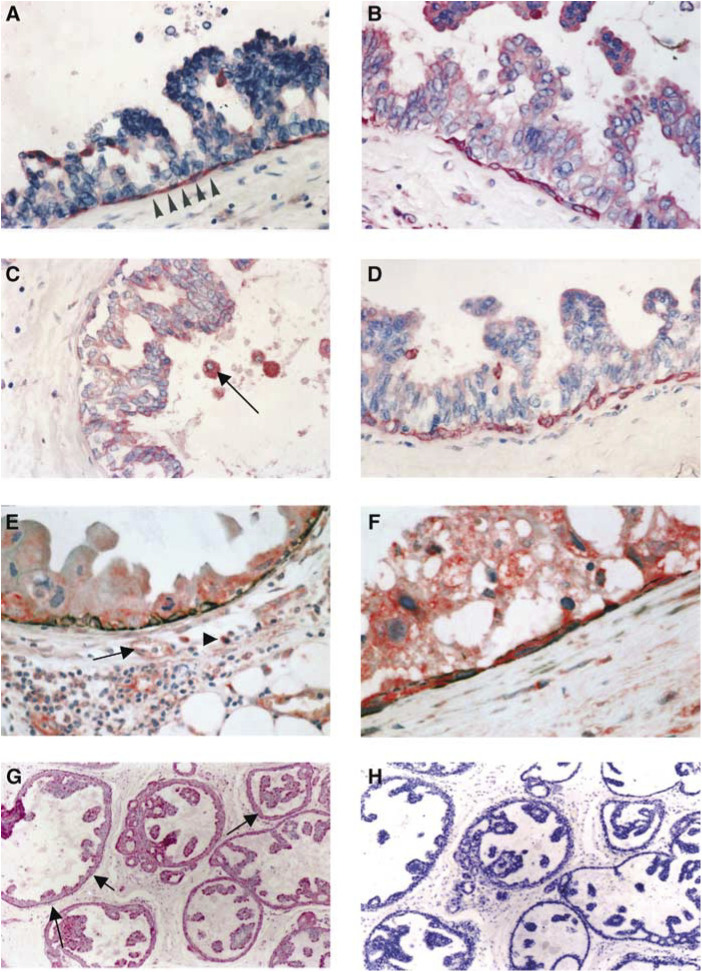
(**A–D**) Represent one intermediate-grade DCIS stained with mAb anti-uPA (**A**), anti-PAI-1 (**B**) and anti-uPAR IID7 (**C**, **D**). (**A**) Myoepithelial cells (arrows) show a positive anti-uPA immunoreaction, tumour cells show only a faint reaction; (**B**) same DCIS as in image B, showing an incomplete ME layer with a strong anti-PAI-1 immunoreaction; tumour cells show a faint immunoreaction. (**C**, **D**) Same DCIS as in images A and B stained with mAb anti-uPAR IID7; the ME layer in image C is absent and the tumour cells are partly detached from the bm; MEs in image D show a strong anti-uPAR immunoreaction; tumour cells in both images are weakly anti-uPAR positive; luminal macrophages (arrow) in image C strongly express the uPAR antigen. (**E**) Represents a high-grade DCIS double stained for anti-PAI-1 (red colour) and anti-SMA (black colour); MEs are positive for both anti-PAI-1 and anti-SMA, tumour cells, stromal cells (arrowhead) and endothelial cells (arrow) strongly express the PAI-1 antigen. (**F**) Represents a high-grade DCIS double stained for anti-uPAR IID7 (red colour) and anti-SMA (black colour); MEs are positive for both anti-uPAR and anti-SMA; tumour cells and stromal cells strongly express the uPAR antigen. (**G**, **H**) *In situ* hybridisation using fluorescein-labelled oligodeoxynucleotides complementary to uPAR-mRNA in a non-high-grade DCIS (grade 1); (**G**) antisense probe: a distinct reaction in MEs, tumour cells, stromal cells and endothelial cells is observed; (**H**) no reaction is seen with sense probe.

). Likewise, the DCIS were screened for the reactivity of tumour cells with the various antibodies; mAb IID7-stained tumour cells in the same 56 specimens (18 grade 1; 14 grade 2; 24 grade 3) in which MEs showed a positive reaction. In 50 specimens (15 grade 1; 15 grade 2; 20 grade 3) within this group tumour cells were stained using pAb HU277. In 31 of those cases (11 grade 1; 10 grade 2; 10 grade 3) tumour cells showed positive immunoreactions using mAb #3936. In all of the cases, fibroblasts and macrophages were stained to various degrees by any of the antibodies. Endothelial cells showed a positive immunoreaction in 56 specimens (17 grade 1; 16 grade 2; 23 grade 3) using mAb IID7 and in 38 cases (14 grade 1; 10 grade 2; 14 grade 3) using pAb HU277. With mAb #3936, no reaction of endothelial cells was seen at all.

In all 12 normal (non-tumour) breast tissue specimens, macrophages and fibroblasts were stained by any of the antibodies (see Table 1b). In 10 of the specimens, MEs were stained by mAb IID7, eight specimens of those reacted with HU277 and five of those with mAb #3936. In 10 of the tissue sections, normal epithelial cells stained with mAb IID7 (Figure 1D), and in seven cases, epithelial cells showed a positive immunoreaction using pAb HU277. In only four cases, epithelial cells were positive with mAb #3936. Endothelial cells of normal breast tissue stained with mAb IID7 (7 specimens) and pAb HU277 (4 specimens).

In 20 cases of high-grade DCIS and in eight cases of normal breast tissue, a double immunostaining was performed using mAb IID7 and anti-calponin. In all cases, MEs showed a positive immunoreaction with both antibodies (Figures 1E and 2F).

**Figure 3:**
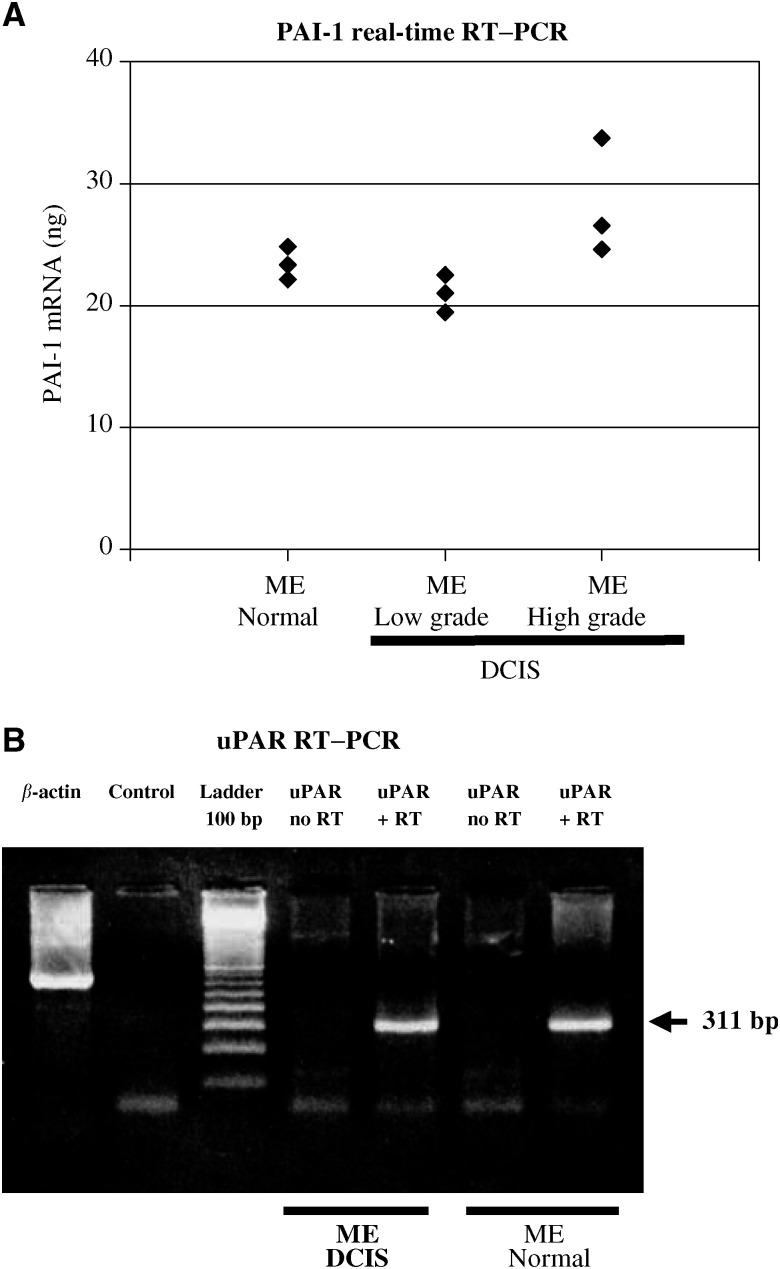
(**A**) Quantitative RT–PCR of mRNA derived from microdissected MEs of normal breast tissue, low- and high-grade DCIS using PAI-1-specific primers. From each group one case was selected and a LightCycler™ analysis was performed in triplicate. The amount of PAI-1 mRNA was calculated by assorting each CP to a standard curve. (**B**) The RNA of MEs (DCIS and normal breast tissue) was isolated, reverse transcriptase reaction followed by a PCR using uPAR primers (see Materials and methods) was performed. The RT–PCR reveals a 311 bp product in both probes (DCIS and normal breast tissue); without RT reaction no product was received.

In 15 specimens of DCIS (five grade 1; five grade 2; five grade 3; all anti-uPAR positive) and in eight cases of normal breast tissue frozen sections and anti-SMA immunoreactions were performed followed by a laser capture microdissection of the MEs. RNA from 1500 to 2000 MEs of each case was isolated and an RT–PCR was performed. In all cases, the expected 311 bp PCR product was obtained (Figure 3B

). In addition, we have performed PAI-1-specific real-time PCR on RNA samples derived from ME cells of normal breast tissue, low- and high-grade DCIS. Each experiment was performed in triplicate. Comparison of the calculated mRNA amounts in each group revealed only slight changes in PAI-1 mRNA (ME_normal breast tissue_: 22.12–24.8 ng; ME_low-grade DCIS_: 19.46–22.48 ng; ME_high-grade DCIS_: 24.58–33.72 ng).

Furthermore, we have studied the PAI-1 expression by immunohistochemistry and *in situ* hybridisation of 60 DCIS and 12 specimens of normal breast tissue. Myoepithelial cells stained with mAb PAI-1 in 34 cases of DCIS (Figure 2E), of those 24 specimens were grade 3, eight specimens were grade 2 and two cases were grade 1. In 56 DCIS (19 grade 1; 14 grade 2; 23 grade 3), a positive immunoreaction of tumour cells was observed (Figure 1C). In all cases, macrophages, fibroblasts and endothelial cells stained with mAb PAI-1. In only one specimen of normal breast tissue MEs and epithelial cells showed a weak positive anti-PAI-1 immunoreaction. In all normal breast tissue specimens stromal cells and endothelial cells stained with mAb PAI-1 (Figure 1F). All tumour tissue sections were probed for the presence of PAI-1 mRNA by *in situ* hybridisation using fluorescein-labelled oligodeoxynucleotides. With no exception, macrophages, fibroblasts and endothelial cells showed a positive reaction with the antisense probe. Myoepithelial cells showed positive reactions in 34 cases (two grade 1; eight grade 2; 24 grade 3) and tumour cells in 56 cases (18 grade 1; 15 grade 2; 23 grade 3) (Figure 1G and H). In 15 high-grade DCIS anti-PAI-1-/anti-calponin double immunostaining was performed. In all high-grade DCIS, a double staining (anti-PAI-1: red colour; anti-calponin: black colour) of MEs was observed (Figure 2E).

**Figure 4:**
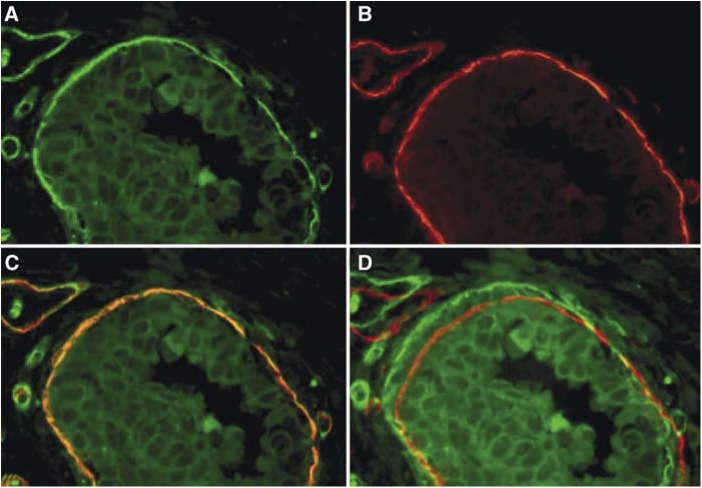
Immunofluorescence double staining of a high-grade DCIS with mAb anti-Vn ((**A**) red signal) and anti-collagen type-4 ((**B**) green signal); (**C**) both immunoreactions are associated within the bm of the breast duct and of a blood vessel; (**D**) DCIS double immunolabelled for Vn and collagen type-4 with a 10-pixel shift of the red signal (collagen type-4), revealing clearly a green and red signal in the bm's.

Double staining with anti-Vn and anti-collagen type-4 mAbs revealed an association of both proteins in bm's of breast ducts and blood vessels in all examined DCIS cases (*n*=10) (Figure 4

). In eight cases, the tumour cells and stroma showed a weak positive anti-Vn immunoreaction.

## DISCUSSION

Two epithelial cell types line the entire normal duct and lobular system of the human breast. There is an inner ‘luminal’ cell layer and an outer ME layer. The MEs of the breast ducts and breast glands may play a special role concerning the invasion of tumour cells because of their important anatomic location between the bm and the epithelial cells/noninvasive tumour cells of DCIS. The ME, which lies on the epithelial side of the bm, is thought to contribute considerably to both the synthesis and remodelling of this structure. The ME also lies in direct juxtaposition to normally proliferating and differentiating cells in healthy breast tissue and to abnormally proliferating and differentiating epithelial cells in precancerous lesions of the breast (Lakhani and O'Hare, 2001). In this study, we have demonstrated by multimodal methods that uPAR protein and mRNA is expressed in most MEs of the normal duct and lobular system and in DCIS. This is an important issue to address since the uPA system plays an important role in matrix degradation and invasion. In addition to promoting cell migration by focusing uPA proteolytic activity to the cell surface, uPAR can physically be associated with another ligand, Vn, mediating cell adherence to the extracellular matrix such as bm. We have demonstrated by double staining that the extracellular matrix protein Vn and collagen type-4 are colocalised in the bm of DCIS and normal breast ducts/acini. In Figure 4 (double staining of a DCIS using anti-collagen type-4 and anti-Vn mAb), one can see that both proteins are associated within the bm of a breast duct and vessel walls. This is no surprising result, since a strong interaction of Vn and collagen type-4 *in vitro* is well known (Gebb *et al*, 1986). Furthermore, a colocalisation of both proteins is known in the bm of the kidney tubulus and of vascular bm's (Falk *et al*, 1987; Sawa *et al*, 1993). The presence of Vn within the bm and the expression of uPAR in MEs of both DCIS and normal breast glands suggests an important cell–matrix interaction, which regulates the cell adhesion and detachment. uPA is the physiological activator of this ‘Vn’ receptor, which means that uPA stabilises the Vn-uPAR binding and thereby the cell–matrix contact. PAI-1 is not only a protease inhibitor but also resolves the Vn-uPAR binding and releases the cells from the cell–matrix contact (Wei *et al*, 1996). Therefore, uPAR in MEs of the breast may play a multifunctional role. In the normal breast tissue uPAR is necessary for the physiological shedding of epithelial and MEs. By focusing the proteolytic enzyme uPA on the cell surface, uPAR of MEs take part in the remodelling of the bm.

In our study, the MEs express PAI-1 in all high-grade DCIS (*n*=24), whereas anti-PAI-1 immunoreaction of MEs in non-high-grade DCIS without comedo-type necrosis (VNs group I) was found in only two of 20 cases and in non-high-grade DCIS with comedo-type necrosis (VNs group II) in eight of 16 cases. Analysing total RNA of MEs derived from one low- and one high-grade DCIS and one case of normal breast tissue by real-time RT–PCR exhibited no significant differences in PAI-1 expression (Figure 3A). One case with anti-PAI-1 immunoreaction of MEs was observed in normal breast ducts/glands. On the contrary, uPA expression was found in nearly all (11 of 12) MEs of the normal breast tissue and only in six of 24 cases in MEs of high-grade DCIS, whereas the tumour cells of the high-grade DCIS showed a positive anti-uPA immunoreaction in 18 of 24 cases. These results seem to us as the expression of uPA and PAI-1 in MEs of high-grade DCIS and in MEs of normal breast tissue is inversely regulated, whereas the uPAR expression of MEs is relatively constant in all examined lesions.

In one case of intermediate-grade DCIS, a few ducts showed an incomplete ME layer expressing uPAR and PAI-1 (Figure 2A–D). In this case, a few ducts had a complete loss of the ME layer. A moderate anti-uPA immunoreaction of MEs was found. In this interesting case, we speculate that high levels of PAI-1 and low levels of uPA in MEs are involved in the Vn-mediated detachment of the MEs.

A detailed analysis by Sternlicht and Barsky (1997) revealed that MEs tend to express low levels of matrix-degrading proteinases (e.g. matrix metalloproteinases-2 and -9 and uPA), but relatively high levels of proteinase inhibitors (tissue-inhibitor metalloproteinase-1, protease nexin II/*β*-amyloid precursor protein, PAI-1) and the tumour suppressor maspin. They conclude that MEs regulate the progression of DCIS to invasive cancer by inhibiting cell invasion. Sternlicht and Barsky (1997) suggest that PAI-1 does not contribute to the anti-invasive phenotype of MEs or, conversely, to a highly invasive and metastatic phenotype of tumour cells. In fact, PAI-1 has been correlated directly with uPA expression and poor prognosis in breast cancer (Schmitt *et al*, 1997). This assumption is in accordance with our findings since in our study PAI-1 expression of MEs was found in all high-grade DCIS.

It is possible that the uPA, PAI-1 and uPAR expression of MEs in DCIS is mediated by a paracrine action of tumour cells and that uPA/PAI-1 play an important role in the cell–matrix interaction (cell adhesion/detachment) of MEs. PAI-1 may be an important component in the detachment of MEs, since PAI-1 is able to attenuate the cell–matrix interaction by resolving the uPAR/Vn binding. Although the role of PAI-1 acting as cell detachment factor could not be demonstrated in our study, we speculate that the loss of the anti-invasive ME layer in DCIS may be triggered by PAI-1 and could be an early sign of subsequent tumour cell infiltration.

In normal breast tissue uPAR of MEs may contribute to the remodelling of the bm by focusing uPA proteolytic activity on the myoepithelial surface. uPA, uPAR and PAI-1 in epithelial cells of normal breast tissue may be involved in the physiological shedding of the glands.
